# Clinical challenges and therapeutic strategies in women with endometriosis, deep infiltrating endometriosis, and/or adenomyosis undergoing assisted reproductive technologies: a narrative review

**DOI:** 10.3389/frph.2026.1802958

**Published:** 2026-05-18

**Authors:** Cemil Kaya

**Affiliations:** Private Practitioner Ankara, Türkiye

**Keywords:** adenomyosis, deep infiltartive endometriosis, endometriosis, IVF, pregnancy, surgery

## Abstract

This narrative review explores contemporary therapeutic options and critically assesses surgical results and *in vitro* fertilization-intracytoplasmic sperm injection (IVF/ICSI) outcomes in women affected by endometriosis, deep infiltrating endometriosis (DIE) and/or adenomyosis. A literature search was conducted in PubMed for English-language publications up to September 2025. Out of 350 screened articles, 91 studies fulfilling the inclusion criteria were ultimately included in the analysis. Endometriosis and adenomyosis are important causes of infertility and frequently coexist. Deep infiltrating endometriosis (DIE) represents a severe form and is often associated with adenomyosis. Endometriosis is linked to reduced oocyte yield and embryo quality, whereas live birth rates (LBR) are generally comparable to other infertility etiologies. IVF/ICSI outcomes in adenomyosis remain controversial, although most studies and meta-analysis report negative effects, particularly in diffuse adenomyosis involving the junctional zone (JZ), enlarged uterus, and severe disease. Evidence regarding gonadotropin-releasing hormone (GnRH) analogue treatment and fresh vs. frozen embryo transfer (FET) cycles is inconsistent; FET appears beneficial in endometriosis but shows similar outcomes to fresh cycles in adenomyosis. When both conditions coexist, adenomyosis has a greater adverse impact on miscarriage and LBR. Cumulative LBR better reflects ART outcomes in these patients. Non-surgical treatments show promise in adenomyosis, and in asymptomatic DIE, first-line IVF/ICSI is reasonable. Both conditions increase pregnancy complications; therefore, pre-ART screening with ultrasonography (US) and magnetic resonance imaging (MRI) using standardized criteria is recommended.

## Introduction

### Endometriosis

Endometriosis is a prevalent, chronic, systemic inflammatory disorder characterized by severe pain and compromised fertility. Its complex pathogenesis involves multiple mechanisms, including retrograde menstruation, cyclic tissue injury and repair, recruitment of endometrial stem cells, Müllerian remnant activation, coelomic metaplasia, lymphatic and hematogenous spread, genetic and epigenetic dysregulation, and alterations in the reproductive tract microbiome. Infertility associated with endometriosis may result from anatomical distortion of the pelvis, cytokine-driven sperm DNA damage and impaired motility, microbial dysbiosis, reduced ovarian reserve, and dysregulation of ovulation, fertilization, and embryo implantation.

While endometriosis affects approximately 10%–15% of women in the general population, its prevalence increases to nearly 50% among infertile women. Moreover, superficial and deep dyspareunia contribute significantly to sexual dysfunction in affected patients. A markedly increased familial risk has been observed, with first-degree relatives exhibiting a sixfold higher likelihood of severe disease (1). Consistent with a molecular contribution to disease severity, Orr et al. (2) demonstrated an association between KRAS mutations and more extensive anatomical involvement.

Three main phenotypes of endometriosis have been identified: ovarian endometrioma (OMA), superficial peritoneal endometriosis (SUP), and DIE (3). The gold standard for the diagnosis of pelvic endometriosis remains laparoscopic biopsy with histological confirmation. US is the cornerstone imaging modality for the evaluation and diagnosis of endometriosis, while MRI demonstrates high diagnostic accuracy, particularly for DIE. Disease severity is commonly assessed using classification systems such as the revised American Society for Reproductive Medicine (rASRM) score and the ENZIAN classification. The ENZIAN system specifically evaluates lesion size, localization, and depth of infiltration. However, the clinical value of currently available staging systems, including rASRM, is limited, as they poorly predict the likelihood of spontaneous or assisted pregnancy and show minimal correlation with symptom severity (4). This narrative review provides a comprehensive overview of current management strategies and evaluates surgical outcomes alongside *in vitro* fertilization (IVF) results in patients with endometriosis, deep infiltrating endometriosis and/or adenomyosis,.

## Materials and methods

A literature search was conducted in PubMed for English-language publications up to September 2025. A narrative review design was adopted. The following combinations of search terms were used: “endometriosis AND IVF or ICSI”, “adenomyosis AND IVF”, “deep endometriosis AND IVF or ICSI”, “endometriosis AND surgery and IVF”, “assisted reproductive technology” “endometrioma” “adenomyosis AND frozen embryo transfer”, “fresh embryo transfer” “adenomyosis AND IVF stimulation”, “endometriosis AND frozen embryo transfer”, “endometriosis AND obstetric outcomes”, AND “adenomyosis AND perinatal outcomes”. The eligibility assessment was conducted in two stages; initially, titles and abstracts were screened, followed by a detailed evaluation of the full texts of potentially eligible studies in terms of methodological adequacy and content relevance. In addition, the reference lists of the included studies were screened to identify additional publications relevant to the topic. The primary objective was to review the current literature regarding the role of IVF/ICSI and surgical management in women with endometriosis, deep endometriosis, and/or adenomyosis, and to assess the associated risks during pregnancy. Studies were eligible for inclusion if they met the following criteria: (1) retrospective or prospective studies or meta-analyses involving reproductive-aged women with endometriosis and/or adenomyosis undergoing IVF/ICSI and/or embryo transfer, and (2) systematic reviews. Limitations were imposed on the English language and on studies involving human subjects. Studies were excluded if they: (1) were case reports, case series, conference abstracts, or expert opinion articles; (2) did not include patients with endometriosis and/or adenomyosis; or (3) did not involve patients undergoing IVF/ICSI. No attempts were made to identify abstracts submitted to scientific meetings. Only studies that provided robust and unbiased data on specific steps of the IVF/ICSI procedure were included in the analysis. A total of 350 articles were screened, and 91 studies that met the eligibility criteria were included in the final evaluation.

## Medical treatment and ART results

### Medical treatment

All currently available medical treatments, including GnRH-a, oral contraceptives, and progestins, have not been shown to improve natural fertility. While medical therapy may reduce lesion size and alleviate pain, it does not enhance spontaneous conception rates. Suppressive hormonal treatments may be considered following endometriosis surgery to control endometriosis-related symptoms and reduce the risk of disease recurrence while awaiting ART (5). In line with the 2022 European Society of Human Reproduction and Embryology (ESHRE) guidelines, hormonal suppression is not recommended as a strategy to improve natural fertility in women with endometriosis (6).

### In endometriosis ART

According to the ESHRE guidelines, ART, including intrauterine insemination (IUI) and IVF/ICSI, are recommended for the management of endometriosis-related infertility (6). Controlled ovarian hyperstimulation (COH) combined with IUI is suggested as a first-line treatment for women with ASRM stage I–II endometriosis who are younger than 35 years, have normal semen parameters, patent fallopian tubes, and adequate ovarian reserve. IVF/ICSI may help overcome several endometriosis-related factors, particularly the inflammatory environment within the pelvic cavity that can negatively affect fertility. It is estimated that IVF/ICSI is required in approximately 10%–25% of women with endometriosis (6, 7). The principal indications for ART include tubal factor infertility, male factor infertility, a low Endometriosis Fertility Index (EFI), and failure of previous treatment approaches.

A systematic review by Kuan et al. (8) reported no significant differences in clinical pregnancy rates (CPR) or LBR between long GnRH agonist and GnRH antagonist protocols. In contrast, a meta-analysis by Han et al. (9) demonstrated that a FET strategy was associated with higher CPR (OR: 1.2), LBR (OR: 1.3), and implantation rates (OR: 1.2) compared with fresh embryo transfer (ET). Furthermore, a recent Cochrane Review concluded that the benefit of ultra-long GnRHa agonist pretreatment (minimum duration of 3 months) prior to IVF or ICSI remains uncertain with respect to CPR, miscarriage rates, and LBR (10, 11). Additionally, a study by Khalifa et al. (12) reported that dienogest had a comparable effect to the ultra-long GnRH agonist protocol, particularly in terms of LBR, in women with endometriosis undergoing IVF (6).

### Oocyte/embryo effect

Data from the Human Fertilisation and Embryology Authority (HFEA) indicate no significant difference in LBR between fresh and FET cycles in women with endometriosis (13). Studies comparing euploidy and aneuploidy rates between women with endometriosis and age-matched controls have reported comparable euploidy rates across groups (14). Moreover, endometriosis does not appear to negatively affect oocyte morphology in IVF/ICSI cycles (15). Although the presence of OMA may reduce oocyte yield in women with endometriosis undergoing IVF/ICSI, it does not seem to adversely affect oocyte quality (16). Similarly, no differences have been observed in the proportion of meiotic abnormalities in *in vitro*–matured oocytes obtained from women with endometriosis compared with controls following COH (17). In line with these findings, a recent meta-analysis including 22 studies demonstrated that endometriosis does not impair embryo morphology, with comparable rates of high-quality embryos and cleavage rates between groups (18). Furthermore, when euploid blastocysts were transferred in hormone replacement therapy (HRT)–FET cycles, LBR were similar between women with endometriosis and controls, suggesting that endometriosis *per se* does not compromise oocyte quality (19). Hovewer, the literature presents mixed results, with several studies reporting modifications in embryo kinetics and reduced viable pregnancy outcomes in women with endometriosis (7). Collectively, the effect of endometriosis on oocyte and embryo quality remains incompletely understood. However, most current studies are limited by small sample sizes; therefore, larger well-designed studies are required to clarify the underlying mechanisms. Although reduced oocyte quality and maturation capacity have been reported, embryo quality appears largely comparable to controls, despite fewer retrieved mature (MII) oocytes in women with endometriomas (6, 9, 19).

### Fertilization/implantation

Several studies have reported conflicting findings regarding fertilization rates in women with endometriosis. Fertilization rates have been shown to be lower in women with ASRM stage I–II endometriosis compared with those with severe endometriosis or tubal factor infertility (20). Harb et al. (21) reported an approximately 7% reduction in both fertilization rates and CPR in women with mild as well as moderate-o-severe endometriosis.

Consistently, a recent meta-analysis demonstrated that ASRM stage I–II endometriosis is specifically associated with impaired fertilization (OR: 0.77, 95% CI: 0.63–0.93) and early implantation processes (OR: 0.76, 95% CI: 0.62–0.93), whereas more advanced disease negatively affects all stages of reproduction (22). In contrast, a retrospective study evaluating 503 IVF cycles reported no significant difference in fertilization rates when comparing women with endometriosis to those with infertility due to other causes (23). Similarly, a meta-analysis by Mappa et al. (24), including eight studies with a total of 5,661 women in the study group and 62,972 in the control group, found no significant difference in fertilization rates between groups. Overall, these findings suggest that fertilization may be selectively compromised in milder forms of endometriosis, while results remain inconsistent across studies. A meta-analysis by Harb et al. (21) reported an 11%–17% reduction in implantation rates in women with ASRM stage III–IV endometriosis. Similarly, a meta-analysis by Qu et al. (22) demonstrated a significantly lower implantation rate in women with endometriosis compared with controls (*p* = 0.04), although no significant differences were observed in overall reproductive outcomes. Regarding adjunctive interventions, studies evaluating endometrial scratching prior to IVF have shown no significant improvement LBR in women with endometriosis compared with controls (25). Furthermore, endometrial receptivity analysis revealed no significant differences in the expression of 238 genes associated with endometrial receptivity between women with and without endometriosis (26). In support of preserved endometrial function, Bishop et al. (19) assessed implantation outcomes in three populations undergoing euploid FET following IVF/ICSI for different indications, including endometriosis, male factor infertility, and preimplantation genetic testing for monogenic disorders, and found no differences in implantation or LBR among the groups. Similarly, another study examining the role of endometrial receptivity in endometriosis- associated infertility reported comparable CPR and LBR in FET cycles using euploid embryos between women with endometriosis undergoing IVF/ICSI and control patients (27). Evidence shows that in FET cycles with high-quality embryos, endometrial receptivity is not significantly impaired in women with endometriosis (19, 27). However, the inherent limitations related to the retrospective design of these studies must be acknowledged. In addition, it should be taken into account that evaluating endometrial receptivity remains highly challenging, and a clear definition of what constitutes normal receptivity has yet to be established. Accordingly, the reduced implantation rates observed in women with endometriosis may be more strongly associated with compromised oocyte and embryo quality than with defects in endometrial receptivity. In cases where adenomyosis coexists with endometriosis, implantation may be compromised, potentially leading to elevated miscarriage rates. Collectively, emerging data suggest that endometriosis is a heterogeneous disease rather than a single entity, while the exact mechanisms connecting it to infertility remain to be elucidated.

### Clinic pregnancy/live birth rate

With respect to favorable IVF/ICSI outcomes, a meta-analysis by Mappa et al. (24) demonstrated that endometriosis does not significantly affect LBR or CPR compared with other causes of infertility. Similarly, data from the Human Fertilisation and Embryology Authority (HFEA) reported no significant differences in LBR between women with endometriosis undergoing IVF/ICSI and those with other infertility diagnoses, in both fresh and FET cycles (13). A Norwegian retrospective study also reported comparable cumulative LBR between women with endometriosis and controls (66.0% vs. 66.7%), and the 2022 Society for Assisted Reproductive Technology (SART) report found no significant differences in IVF/ICSI outcomes between women with endometriosis and control populations (28, 29).

In contrast, several studies have reported adverse IVF/ICSI outcomes, particularly in women with advanced disease. Horton et al. (30) observed a 12% reduction in LBR following IVF/ICSI, which was confined to patients with ASRM stage III–IV endometriosis. Consistent with these findings, Liao et al. (31) demonstrated a significant reduction in LBR in women with endometriosis undergoing IVF/ICSI compared with those undergoing IVF for other indications.

A large meta-analysis by Paffoni et al. (32), including 137,182 cycles from SART and 24,900 cycles from HFEA, analyzed 7,212 oocyte donation cycles and identified a significantly lower LBR in recipients with endometriosis (OR: 0.89; 95% CI: 0.81–0.97), suggesting a potential uterine or systemic effect of the disease. Harb et al. (21) conducted a meta-analysis of 27 observational studies involving women with ASRM stage I/II and III/IV endometriosis undergoing IVF/ICSI. Their analysis demonstrated a 7% reduction in fertilization rates and CPR across all ASRM stages, while women with stage III–IV disease experienced a more pronounced reduction in LBR (14%; RR: 0.79; 95% CI: 0.69–0.91), along with lower implantation rates (RR: 0.86; 95% CI: 0.68–1.08). Similarly, a meta-analysis by Hamdan et al. (33), including 17 studies and 17,593 IVF cycles, reported a significant decrease in LBR (30%) and CPR (40%) in women with ASRM stage III–IV endometriosis (OR: 0.78; 95% CI: 0.65–0.95). Importantly, in the same analysis, women with ASRM stage I/II endometriosis demonstrated LBR comparable to those of women without endometriosis.

These findings are further supported by meta-analyses by Rossi et al. (34) and Morcel et al. (35), which reported similar CPR in women with ASRM stage I/II endometriosis compared with controls, whereas significantly lower cumulative CPR and LBR were observed in women with ASRM stage III/IV disease. Collectively, these data suggest that while overall IVF/ICSI outcomes in women with endometriosis are broadly comparable to those observed in other infertility diagnoses (13, 24, 28, 29), advanced-stage endometriosis is consistently associated with reduced implantation and LBR, whereas mild disease appears to have minimal impact on IVF success (Table 1).As results, growing evidence indicates that endometriosis represents a spectrum of related yet distinct conditions characterized by diverse pathophysiological mechanisms and clinical phenotypes. Given the progressive staging of endometriosis from minimal to severe disease, studies that do not stratify participants according to disease stage may underestimate stage-specific effects. Advance stages of endometriosis are linked to increased gonadotropin needs and reduced reproductive success. To improve methodological robustness, future investigations should incorporate uniform staging criteria when examining the influence of endometriosis severity on clinical outcomes. This heterogeneity may differentially affect female fertility, contributing to variability in disease severity, therapeutic response, and reproductive outcomes. Improvements in IVF treatment modalities, protocols and laboratory conditions will be reflected in the results over time. The effect of endometriosis on LBR following IVF/ICSI remains contentious. While some studies report diminished outcomes, potentially related to impaired oocyte quality, embryo development, and endometrial receptivity, others demonstrate comparable results after adjustment for age and ovarian reserve (21, 28, 29, 31, 33).

**Table 1:** The characteristics of selected studies related to ART outcomes with endometriosis.

Reference	Intervention and/or study type	Comparable and/or positive IVF/ICSI outcomes	Uncertain and/or negative IVF/ICSI outcomes	Conclusion and/or recommendations
Kuan et al. (8)	Systematic review/GnRH agonist vs. antagonist outcomes	No significant differences in CPR or LBR		The latest ESHRE guidance does not recommend ultra-long GnRH agonist before IVF/ICSI
Han et al. (9)	Systematic review and meta-analysis/ FET vs. Fresh cycle outcomes	The FET strategy yielded higher clinical pregnancy (OR: 1.25; 95% CI: 1.11, 1.40), live birth rates (OR: 1.31; 95% CI: 1.15, 1.49), and implantation rates (OR: 1.27; 95% CI: 1.05, 1.54) compared to the fresh ET strategy.		FET strategy has been associated with more favorable reproductive outcomes compared to the fresh ET strategy in women with endometriosis
Cochrane Review (11)	Ultra-long GnRH agonist		Uncertain with regards to CPR, miscarriage, and LBR	Not recommended
Kamath et al. (13)	HFEA anonymized data from 1996 to 2016. This comprised of a total of 758 donor oocyte recipients, where the recipients were women diagnosed with endometriosis, and 12,856 autologous IVF cycles where the women were diagnosed with endometriosis as the sole cause of infertility.	There was no significant difference in the LBR in women with endometriosis undergoing donor oocyte recipient fresh embryo transfer cycles compared to women undergoing autologous IVF fresh embryo transfer cycles [31.6% vs. 31.0%; odds ratio (OR) 1.03, 99.5% CI: 0.79–1.35].		Endometriosis does not compromise embryo quality from the perspective of morphology.
Juneau et al. (14)	305 patients with endometriosis who produced 1,880 blastocysts and PGS	Aneuploidy rates equivalent to their age-matched peers in IVF population who do not have endometriosis.		Aneuploidy rates equivalent to their age-matched peers in IVF population who do not have endometriosis.
Robin et al. (15)	A total of 596 women treated for IVF-ICSI, retrospective study	No difference in AOQI and MOMS scores was found between endometriosis and control women (adjusted *p* = 0.084 and 0.053, respectively).		Endometriosis does not have a negative impact on oocytes' morphology in IVF-ICSI.
Barcelos et al. (17)	To evaluate the meiotic spindle and the chromosome distribution of *in vitro* mature oocytes from stimulated cycles of infertile women with endometriosis, and with male and/or tubal infertility factors	There was no significant difference in the IVM rates between the two groups evaluated (45.6 and 54.5% for the Endometriosis and Control Groups, respectively).		Endometriosis does not increase meiotic abnormality.
Dongye et al. (18)	A systematic review and meta-analysis (22 studies)	Compared with the control group, women with endometriosis had a similar high-quality embryo rate (RR = 1.00; 95% CI, 0.94–1.06), a comparable cleavage rate (RR = 1.00; 95% CI, 0.97–1.02), and a similar embryo formation rate (RR = 1.10; 95% CI, 0.97–1.24). In women with stage III–IV endometriosis, there was no statistically significantly difference in high-quality embryo rate (RR = 1.02; 95% CI, 0.94–1.10), cleavage rate (RR = 1.00; 95% CI, 0.98–1.02)		Endometriosis does not compromise embryo quality from the perspective of morphology.
Bishop et al. (19)	Retrospective cohort study	Euploid FET-ET cycles, LBR, CPR, no difference endometriosis vs. control group		LBR are not affected by endometriosis after euploid blastocysts in HRT FET-ET cycles
Harb et al. (21)	Systematic review and meta-analysis (27 observational study)		Fertilisation rates were reduced in stage I/II of endometriosis (relative risk [RR] = 0.93, 95% confidence interval [95% CI] 0.87–0.99, *p* = 0.03). There was a decrease in the implantation rate (RR = 0.79, 95% CI: 0.67–0.93, *p* = 0.006) and clinical pregnancy rate (RR = 0.79, 95% CI: 0.69–0.91, *p* = 0.0008) in women with stage III/IV endometriosis undergoing IVF treatment.	Endometriosis ASRM III/IV is associated with poor implantation and CPR.
Qu et al. (22)	Systematic review and meta-analysis (70 study)	Implantation rate (*p* = 0.04) and the number of oocyte retrieved (*p* < 0.00001) were significantly lower in women with endometriosis	All other major reproductive and obstetric outcomes were similar in women with and without endometriosis.	Endometriosis have similar reproductive outcomes, but women with endometriosis who conceived after ART are in high risk pregnancy.
Mappa et al. (24)	Systematic review and meta-analysis(40 studies/8,970 women with endometriosis and 42,946 control)	There were no significant differences between the endometriosis and control groups in terms of LBR (OR 1.03, 95% CI: 0.75–1.41, *p* = 0.84), CBR (OR: 0.86, 95% CI: 0.72–1.02, *p* = 0.1), or fertilization rate (OR: 0.96, 95% CI: 0.79–1.15, *p* = 0.64).	Endometriosis was associated with a significantly lower implantation rate (OR: 0.85, 95% CI: 0.74–0.97, *p* = 0.02).	Endometriosis significantly negatively affects implantation rates in women undergoing IVF, despite the absence of significant differences in LBR, CPR, and fertilization rates.
van Hoogenhuijze et al. (25)	Meta-analysis(13 RCTs, (12 published, one unpublished) 4,112 participants	There was no significant difference LBR in women with endometriosis compared to controls		Endometrial scratching in clinical practice should be considered with caution
Horton et al. (30)	Systematic review and meta-analysis in Endometriosis and adenomyosis (104 study)		Milder forms of endometriosis were most likely to affect the fertilization (FR OR: 0.77, CI: 0.63–0.93) and earlier implantation processes (implantation rate OR: 0.76, CI: 0.62–0.93). Increased miscarriage in both adenomyosis and endometriosis (OR: 3.40, CI: 1.41–8.65 and OR: 1.30, CI: 1.25–1.35, respectively),	Milder forms of endometriosis were most likely to affect the fertilization.
Liao et al. (31)	Systematic review and meta-analysis (19 studies)	CPR were not significantly different between groups (OR: 1.03)	Decreased total oocytes [mean difference (MD): −2.06],mature oocytes (MD: −2.07) and LBR (OR: 0.87) in endometriosis/ BMI significantly influenced the risk of endometriosis	*Higher BMI is associated with an increased risk of endometriosis, which negatively affects IVF outcomes
				* Surgical treatment before IVF/ICSI does not appear to enhance IVF success and may further compromise LBR.
Hamdan et al. (33)	Systematic review and meta-analysis (36 studies, three were RCTs)	a similar LBR [OR] 0.94, 95% [CI] 0.84–1.06, I = 35%),	a lower CPR (OR: 0.78, 95% CI: 0.65–0.94, I = 66%), a lower mean number of oocyte (1.98, 95% CI −2.87 I = 97%)	ASRM III/IV have a lower LBR and CPR
Rossi et al. (34)	Systematic review and meta-analysis 813 study)	CPR were similar between stage I–II and controls (OR: 0.99; 95% CI: 0.63–1.56)	CPR lower in stage III–IV than controls (OR: 0.45; 95% CI: 0.29–0.70),	ASRM III/IV endometriosis had significantly lower cumulative CPR and ongoing pregnancy rates
Morcel et al. (35)	Retrospective clinical study		ASRM stage III/IV had significantly lower cumulative CPR and ongoing pregnancy rates	ASRM III/IV pregnancy rates are decreasing cumulatively.
Liang et al. (47)	Systematic review and meta-analysis/ART vs. Surgery	Pregnancy rate per patient (OR, 1.47; 95% CI, 0.59 to 3.63), pregnancy rate per cycle (OR, 1.16; 95% CI, 0.45 to 2.99), and live births per patient (OR, 1.66; 95% CI, 0.56 to 4.91) were comparable in DIE patients		First-line surgery and ART can be effective DIE treatments with similar fertility outcomes
Casals et al. (48)	Systematic review and meta-analysis (98 studies) (Surgery for DIE before IVF/ICSI)	The pregnancy rate per patient was 1.84 (95% confidence interval [CI], 1.28–2.64), the pregnancy rate per cycle was 1.84 (95% CI, 1.26–2.70), and the LBR per patient was 2.22 (95% CI, 1.42–3.46) times more likely for operated patients than for nonoperated ones.		A statistically significant benefit for surgery before IVF/ICSI in patients with DIE endometriosis
Bourdon et al. (50)	Systematic review and meta-analysis (19 studies) (Surgery for DIE before IVF/ICSI)		The LBR per cycle was significantly reduced in the case of surgical treatment before IVF/ICSI [0.53[0.33, 0.86]; *I*^2^ = 30%)	The LBR per cycle was significantly reduced in the case of surgical treatmet before IVF/ICSI

ART, assisted reproductive treatment; HFEA, human fertilization and embryology authority; PGS, preimplantation genetic screening; AOQI, average oocyte quality index; MOMS, metaphase II oocyte morphological scoring system. (MOMS); CPR, clinical pregnancy rate; LBR, live birth rate; FET, frozen embryo transfer; ET, embryo transfer; FET, frozen embryo cycles; DIE, deep infiltrative endometriosis; IVF, *In vitro* fertilization; ICSI, intra-cytoplasmic sperm injection.

### In SUP/surgery vs. ART

According to the 2022 ESHRE guidelines, routine surgical intervention prior to ART is not recommended for women with ASRM stage I–II endometriosis, as no clear benefit on LBR has been demonstrated. Nevertheless, evidence regarding the impact of surgery on reproductive outcomes remains mixed. A recent meta-analysis reported a significant increase in CPR following operative laparoscopy for endometriosis compared with placebo or diagnostic procedures alone (OR: 1.63; 95% CI: 1.13–2.35) (36). Similarly, the meta-analysis by Hodgson et al. (37) demonstrated improved CPR following surgical treatment in women with endometriosis. Notably, only one meta-analysis to date, conducted by Jin et al. (38), including four trials focusing on SUP, demonstrated a significant improvement in LBR following laparoscopic surgery (RR: 1.52; 95% CI: 1.26–1.84; *p* < 0.01), as well as increased pregnancy rates (RR: 1.44; 95% CI: 1.24–1.68; *p* < 0.01). Nevertheless, the absolute benefit was modest, with an estimated number needed to treat of approximately 12 women to achieve one additional pregnancy. Evidence directly evaluating the impact of surgical intervention prior to IVF/ICSI remains limited. To date, only a single retrospective cohort study has compared reproductive outcomes in women with SUP who underwent complete excision of all visible endometriotic lesions before IVF/ICSI (*n* = 399) with those who underwent diagnostic laparoscopy alone (*n* = 262). In this study, complete surgical excision was associated with significantly higher CPR and LBR (OR: 1.47; 95% CI: 1.01–2.13) compared with diagnostic laparoscopy alone (39). When surgery is indicated for other clinical reasons, such as pain management, operative laparoscopy may therefore be considered as a treatment option in women with infertility associated with ASRM stage I–II endometriosis (6).

### In OMA/surgery vs. ART

Several meta-analyses have demonstrated that ovarian cystectomy for OMA in women undergoing IVF/ICSI does not improve CPR or LBR (16, 33). Consistently, Alseshre et al. (40) reported comparable embryo quantity and quality between women with OMA and control subjects. However, ovarian cystectomy performed prior to IVF/ICSI may be associated with higher cycle cancellation rates due to poor ovarian response, particularly in cases involving larger endometriomas. A reduced response to COH has mainly been observed in women with OMA exceeding 4 cm in diameter. Overall, surgical treatment of OMA does not appear to enhance IVF/ICSI outcomes. Importantly, surgical excision of OMA has been shown to result in a significant decline in anti-Müllerian hormone (AMH) levels, especially in women with bilateral OMA or lesions larger than 5 cm (41). This decline is thought to result from inadvertent removal of healthy ovarian tissue, thermal injury during surgery, and subsequent inflammatory changes. Meta-analyses evaluating the impact of OMA on IVF/ICSI outcomes have consistently reported a reduction in the number of mature oocytes retrieved in women with OMA compared with controls. Nevertheless, no significant differences have been observed in gonadotropin dose or stimulation duration, total number of embryos, proportion of high-quality embryos, CPR, or LBR between women with and without OMA (42). Furthermore, a meta-analysis focusing on postoperative recurrence reported recurrence rates of 4%, 14%, 17%, and 27% at 3, 6, 12, and 24 months following surgery, respectively (43). Evidence shows that OMA surgery does not enhance IVF/ICSI outcomes and should be considered mainly for pain, with careful techniques to preserve ovarian reserve (1, 6, 41).

### DIE/surgery vs. ART

DIE is a severe form of the disease, defined by the infiltration of fibrous and muscular tissues beneath the peritoneal surface to a depth greater than 5 mm. DIE affects approximately 3.8% to 37% of women with endometriosis. DIE is frequently associated with focal external adenomyosis (44). Diagnosis of DIE is based on strict imaging criteria, primarily MRI, and in women with a previous history of endometriosis surgery, histological confirmation (45). Therapeutic strategies mainly include surgical management and ART.

A retrospective cohort study including 222 women with DIE who underwent a total of 440 ART cycles (fresh and FET) evaluated the impact of prior surgery on reproductive outcomes. Of these patients, 155 women (69.8%) had a history of surgical treatment for endometriosis. The cumulative LBR after four IVF/ICSI cycles was 26% in women with a prior history of endometriosis surgery, compared with 51.3% in those without previous surgery (*p* < 0.001). Multivariate analysis identified prior surgery for endometriosis (*p* = 0.001) and previous surgery for OMA (*p* = 0.005) as independent predictors of reduced pregnancy rates. These findings suggest that, in women with DIE, a history of surgical intervention may be associated with less favorable IVF/ICSI outcomes (46).

A meta-analysis by Liang et al. (47) demonstrated that pregnancy rate per patient (OR: 1.47), pregnancy rate per cycle (OR: 1.16), and LBR (OR: 1.66) were comparable in women with DIE treated with either surgery or IVF/ICSI as a first-line approach. When both complete and incomplete surgical excision procedures were included, surgical management was associated with a significant increase in pregnancy rate per patient (OR: 1.63; 95% CI: 1.11–2.40).

Similarly, a meta-analysis by Casals et al. (48) compared reproductive outcomes in women who underwent surgery for DIE prior to IVF with those who proceeded directly to IVF without prior surgical intervention. In this analysis, women who underwent DIE surgery before IVF had higher pregnancy rates per patient (OR: 1.84) and were more than twice as likely to achieve a LBR per patient (OR: 2.22) compared with non-operated women. In contrast, Daniilidis et al. (49) reported no evidence supporting routine surgical excision of DIE prior to IVF/ICSI to improve reproductive outcomes in women with endometriosis.

Consistent with these findings, a meta-analysis by Bourdon et al. (50) compared ongoing pregnancy rates and LBR in women who underwent endometriosis surgery before ART with those who underwent first-line IVF/ICSI. After excluding studies with a high risk of bias, LBR per cycle were significantly lower in women who had undergone surgical treatment prior to IVF/ICSI, supporting the conclusions of Daniilidis et al. (49). More recently, a meta-analysis by Riemma et al. (51) found that surgery followed by IVF/ICSI did not significantly improve LBR in women with OMA (OR: 0.89; 95% CI: 0.68–1.16) or DIE (OR: 1.82; 95% CI: 0.70–4.77). Evidence from RCTs does not support the routine use of operative laparoscopy to improve reproductive outcomes in DIE (6, 7, 51). Surgical intervention should be limited to specific indications including obstruction or medically refractory pain (50, 51). Management should be personalized considering patient preference, age, clinical presentation, and the risk of reoperation.

### Adenomyosis

Adenomyosis is characterized by the presence of endometrial-like tissue, composed of glands and stroma, within the myometrium. Two main theories have been proposed to explain its pathogenesis. The first suggests invagination of the endometrial basalis into the underlying myometrium, whereas the second proposes *de novo* development resulting from metaplasia of embryonic Müllerian remnants. Increasing evidence supports a role for somatic mutations, particularly involving KRAS and PIK3CA, in the pathogenesis of adenomyosis (52). Based on MRI findings, Kishi et al. (53) classified adenomyosis into four subtypes according to lesion localization. Diffuse adenomyosis is more prevalent than the focal form. Diffuse disease is more commonly observed in older women, whereas focal adenomyosis tends to occur at a younger age and is more frequently associated with endometriosis. Furthermore, internal adenomyosis is more often associated with uterine fibroids, while external adenomyosis shows a stronger association with endometriosis (51).

The diagnostic accuracy of transvaginal US and MRI for adenomyosis has been shown to be comparable. In the diagnosis of adenomyosis, MRI—similar to transvaginal US—aims to identify the direct imaging features of the disease. On MRI, adenomyosis is strongly suspected when the JZ thickness measures ≥12 mm (54). Histopathological confirmation is not required for the diagnosis of adenomyosis in women undergoing evaluation and treatment for infertility. According to the Morphological Uterus Sonographic Assessment (MUSA) criteria, adenomyosis is diagnosed when at least one direct sonographic feature is present, including myometrial cysts, hyperechogenic islands, or subendometrial lines or buds. Adenomyosis has been reported in approximately 24.4% of young infertile women undergoing IVF/ICSI cycles. Its prevalence is even higher among women with recurrent pregnancy loss and those with a history of previous ART failure, reaching 38.2% and 34.7%, respectively.

Adenomyosis may contribute to infertility through multiple mechanisms, including thickening of the JZ, abnormal uterotubal peristalsis, and biochemical, functional, and epigenetic alterations in both eutopic and ectopic endometrium. Repeated microtrauma at the endomyometrial junction has also been proposed as a contributing factor in the development of adenomyosis. Additional mechanisms potentially involved include anatomical distortion of the uterine cavity, altered sex steroid hormone signaling, increased inflammatory mediators and oxidative stress, reduced expression of implantation markers, decreased expression of adhesion molecules, and dysregulation of genes involved in embryonic development. The extent and subtype of adenomyosis appear to be important determinants of its impact on fertility. The frequent coexistence of adenomyosis with endometriosis may further contribute to impaired reproductive outcomes. Moreover, uterine leiomyomas coexist in approximately 35%–55% of women with adenomyosis (55, 56).

### In adenomyosis ART

IVF outcomes in women with adenomyosis remain heterogeneous, with numerous studies and meta-analyses reporting adverse reproductive results. A meta-analysis by Younes and Tulandi (57) demonstrated a 41% reduction in LBR and an increased risk of miscarriage in women with adenomyosis undergoing IVF/ICSI. Similarly, Horton et al. (30) reported a 55% reduction in LBR following IVF/ICSI in this population. In contrast, Nirgianakis et al. (58) found no significant differences in reproductive outcomes between women with adenomyosis and control subjects and further suggested that the adenomyosis phenotype (focal vs. diffuse) did not significantly influence IVF/ICSI outcomes. More recently, Bourdon et al. (59) conducted a matched cohort study including 285 women with adenomyosis and 285 controls, reporting a significantly lower cumulative LBR in the adenomyosis group compared with controls (41.4% vs. 51.9%; OR: 0.65, 95% CI: 0.47–0.91; *p* = 0.012). CPR were also significantly reduced in women with adenomyosis (53.3% vs. 63.9%; *p* = 0.011). In contrast to these findings, a prospective IVF cohort study evaluating 99 women with adenomyosis and 549 controls undergoing preimplantation genetic testing for aneuploidy followed by FET found no significant differences in CPR, LBR, or miscarriage rates between the two groups (60). In Mavrelos et al. (61) demonstrated that mild forms of adenomyosis have limited impact while more severely affected women have poorer outcomes. In terms of miscarriage, in a meta- analysis by Vercellini et al. (62), the miscarriage rate was more prevalent (RR = 2.1 (95% CI: 1.20–3.75) in women with adenomyosis vs. control (32% vs. 14%). A systematic review by Horton et al. (30) which seven comparative observational studies that involved IVF/ICSI cycles, there was an increase in miscarriages (OR =  3.49, CI: 1.41–8.65, *p* = 0.007; *n* = 6). In patients with adenomyosis undergoing ART in oocyte donation, normal implantation rates and higher miscarriage rates have been reported (63, 64). Furthermore, women with adenomyosis have an increased risk of miscarriage, even using euploid embryos (65) (Table 2).

**Table 2:** The characteristics of selected studies related to ART outcomes with adenomyosis.

References	Intervention and/or study type	Comparable and/or positive IVF/ICSI outcomes	Uncertain and/or negative IVF/ICSI outcomes	Conclusion and/or recommendations
Younes and Tulandi (57)	A meta-analysis		a 41% decrease in LBR and an increased risk of miscarriage in women with adenomyosis.	Adenomyosis has a detrimental effect on IVF/ICSI reproductive outcomes.
Han et al. (9)	Systematic review and meta-analysis/ FET vs. Fresh cycle outcomes	In adenomyosis, the IVF/ICSI outcomes were comparable between the FET and fresh ET strategies.		Comparable pregnancy rates between the two groups (FET vs. Fresh cycle)
Horton et al. (30)	Systematic review and meta-analysis		A 55% reduction in LBR after IVF-ICSI	Adenomyosis has a detrimental effect on IVF/ICSI reproductive outcomes.
Nirgianakis et al. (58)	Systematic review and meta-analysis		A lower CPR (odds ratio [OR] 0.69; 95% confidence interval [CI] 0.51–0.94) and higher miscarriage rate (OR: 2.17; 95% CI: 1.25–3.79) in adenomyosis	Adenomyosis has a detrimental effect on IVF/ICSI reproductive outcomes.
Mavrelos et al. (61)	A prospective multicentre study		A lower clinical pregnancy rate (21/72 [29.2%, 95% CI 18.6–39.6] vs. 129/303 [42.6%, 95% CI: 37.1–48.2], *p* = 0.044, relative risk (RR) 0.68 [95% CI 0.47–1.00]).	Condition severity expressed as a number of morphological features on ultrasound scan increases the magnitude of the effect.
Neal et al. (60)	A prospective cohort study	No difference in the rate of CPR, 1.47 (95% CI, 0.85–2.56)), miscarriage (aOR, 1.3 (95% CI, 0.62–2.72)) or LBR 1.28 (95% CI, 0.78–2.08)) between subjects with and those without adenomyosis.		Routine screening for asymptomatic adenomyosis in an unselected infertile patient population undergoing frozen embryo transfer may not be warranted
Vercellini et al. (62)	Systematic review and meta-analysis (17 studies)		The miscarriage rate was more prevalent (RR 2.1 (95% CI: 1.20–3.75) in women with adenomyosis vs. control (32% vs. 14%).	Adenomyosis has an increased risk of miscarriage
Sachs-Guedj et al. (65)	HRT- FET cycle/Euploid ET		Adenomyosis decreased CPR (aOR 0.62, 95% CI: 0.39–0.98, *p* = 0.040) and LBR (aOR 0.46, 95% CI: 0.27–0.75, *p* = 0.003) and significantly increased the miscarriage rates (aOR 2.13, 95% CI: 0.98–4.37, *p* = 0.045)	Even with euploid embryo transfer, the low rates are high in adenomyosis
Bourdon et al. (67)	A single-center observational study.	FET-ET was associated with significantly higher odds of LBR compared with fresh ET (odds ratio = 1.80; 95% CI = 1.02–3.16).		FET-ET is an attractive option in adenomyosis regardless of different type

HRT, hormone replacement therapy; FET, frozen embryo transfer; ET, embryo transfer; CPR, clinical pregnancy rate; LBR, live birt rate; CI, confidence interval; IVF, *In vitro* fertilization; ICSI, intra-cytoplasmic sperm injection.

Regarding the phenotypic impact of adenomyosis on IVF outcomes, evidence remains conflicting. A retrospective multicenter study evaluating IVF results according to adenomyosis phenotype reported a significantly higher LBR per transfer in women with diffuse adenomyosis compared with those with focal disease (47/166 [28.3%] vs. 9/62 [15%], respectively; OR: 2.32, 95% CI: 1.03–5.78; *p* = 0.034) (53). In contrast, a meta-analysis by Wang et al. (66) demonstrated less favorable reproductive outcomes in women with diffuse and symptomatic adenomyosis. In this analysis, diffuse adenomyosis was associated with a significantly lower LBR (OR: 0.57, 95% CI: 0.34–0.96) and a higher miscarriage rate (OR: 2.48, 95% CI: 1.28–4.82) when compared with focal adenomyosis.

A meta-analysis by Han et al. (9) reported that pregnancy outcomes in women with adenomyosis were comparable between HRT-FET and fresh ET cycles. FET is preferably performed in estradiol- and progesterone-based hormone replacement cycles. In contrast, Bourdon et al. (67) demonstrated that FET was associated with significantly higher cumulative LBR compared with fresh ET (OR: 1.80; 95% CI: 1.02–3.16). A meta-analysis by Ge Li et al. (68) evaluated reproductive outcomes according to COH protocols in IVF/ICSI cycles among women with adenomyosis. In fresh ET cycles involving women aged ≥35 years, CPR were significantly higher with ultra-long and long GnRH agonist protocols compared with antagonist and short protocols (OR: 1.33; 95% CI: 1.06–1.66; *I*^2^ = 40%). These findings suggest that ultra-long or long protocols may confer a reproductive advantage in women with adenomyosis undergoing IVF/ICSI with fresh ET. However, in FET cycles, no significant differences were observed in implantation, CPR, or LBR among embryos derived from different COH protocols. Despite accumulating evidence, the causal relationship between adenomyosis and infertility has not yet been fully established. Most studies exhibit heterogeneity in diagnostic criteria, particularly for adenomyosis, as different imaging modalities such as transvaginal US and MRI—or both—have been used. In addition, subtypes of endometriosis and adenomyosis are often not distinguished, and the inclusion of patients with coexisting disease limits the ability to evaluate their independent effects. Therefore, more high-quality evidence is needed to clarify the impact of adenomyosis on ART outcomes and to optimize management strategies. At present, available evidence is insufficient to formulate firm evidence-based recommendations for the management of infertility in women with adenomyosis.

### GnRH agonist and aromatase inhibitör pretreatment in adenomyosis

A meta-analysis by González-Comadrán et al. (69) found no evidence supporting the benefit of GnRH agonist (GnRHa) downregulation before COH or pretreatment prior to FET in women with adenomyosis undergoing IVF. In contrast, Galati et al. (70) reported that long-term GnRH agonist therapy (≥3 months) was associated with a significant improvement in CPR in IVF/ICSI cycles with fresh ET (OR: 1.49; 95% CI: 1.15–1.92). However, this benefit did not reach statistical significance in women undergoing FET cycles (OR: 1.34; 95% CI: 0.70–2.55). Lan et al. (71) demonstrated that women with diffuse adenomyosis achieved significantly higher CPR (55.3% vs. 37.9%; *p* = 0.025) and LBR (43.4% vs. 25.9%; *p* = 0.019) when treated with an ultra-long GnRH agonist protocol compared with a long protocol. Nevertheless, a meta-analysis by Steinmann et al. (72) concluded that current evidence does not support the superiority of GnRHa pretreatment combined with HRT over HRT alone in women with adenomyosis undergoing FET. Increased aromatase activity within adenomyotic tissue has provided the rationale for the use of aromatase inhibitors (AIs) in symptomatic women with adenomyosis undergoing IVF/ICSI. In a randomized trial, Badawi et al. (73) demonstrated that GnRH agonists and AI_s_ were equally effective in reducing adenomyotic lesions. Similarly, Sharma et al. (74), in a randomized trial, reported that low-dose letrozole may represent an effective pretreatment option for women with symptomatic adenomyosis awaiting IVF/ICSI. However, evidence comparing different IVF stimulation protocols in women receiving AI pretreatment prior to ovarian stimulation remains limited.

### Endometriosis/adenomyosis coexisting

Approximately half of adenomyosis cases are accompanied by endometriosis, which may impact reproductive outcomes and pregnancy course through diverse biological mechanisms. Both conditions are characterized by increased local estrogen production, reduced progesterone receptor expression, and enhanced progesterone resistance within the lesions. Adenomyosis, which frequently coexists with endometriosis, may impair embryo implantation, disrupt uterine function, and increase miscarriage rates. In cases where adenomyosis is associated with DIE, the adenomyotic component is external in up to 96% of patients, whereas intrinsic adenomyosis is observed in approximately 15%.

OMA and DIE are more commonly associated with extrinsic adenomyosis. Furthermore, focal adenomyosis is reported in up to 66% of women with DIE (51, 53, 75). A meta-analysis by Wang et al. (66) demonstrated that women with endometriosis and concurrent adenomyosis have a significantly LBR compared with women with endometriosis alone (OR = 0.44; 95% CI: 0.26–0.75). Sharma et al. (76) were the first to retrospectively evaluate pregnancy outcomes in women with endometriosis accompanied by adenomyosis vs. endometriosis alone, using tubal factor infertility as a control group. In this study, 973 women were categorized into four cohorts: endometriosis only (*n* = 355), endometriosis with adenomyosis (*n* = 88), adenomyosis only (*n* = 64), and controls (*n* = 466). The results demonstrated lower LBRs and higher miscarriage rates in women with combined endometriosis and adenomyosis, as well as in those with adenomyosis alone. Elevated local tumor necrosis factor-alpha (TNF-α) levels were found to correlate with active lesions of both endometriosis and adenomyosis. In women affected by endometriosis and adenomyosis, peri- implantation treatment with a TNF-α inhibitor (adalimumab) was shown to significantly increase CPR in FET cycles (77).

Rees et al. (78) reported that only women with combined adenomyosis and endometriosis exhibited significantly reduced ongoing pregnancy rates (OR: 0.30; 95% CI: 0.17–0.61; *p* = 0.001) and LBR (OR: 0.33; 95% CI: 0.17–0.64; *p* = 0.001) compared with matched male-factor subfertility controls. Similarly, Alson et al. (79) demonstrated that, after stratification by treatment cycle, LBR in women with endometriosis and/or adenomyosis were consistently lower across successive IVF/ICSI cycles. The LBR after the first treatment cycle was 30.7% (RR: 0.69; 95% CI: 0.57–0.84; *p* < 0.001), decreasing to 28.6% after the second cycle (RR: 0.72; 95% CI: 0.54–0.96; *p* = 0.023), and 26.2% after the third cycle (RR: 0.83; 95% CI: 0.54–1.28; *p* = 0.183). In contrast, corresponding LBRs in control populations were 45.1%, 41.4%, and 32.3% for the first, second, and third cycles, respectively. These findings indicate that the presence of adenomyosis significantly reduces LBR in IVF/ICSI cycles among women with endometriosis (Table 3). More recently, Zhu et al. (80) reported a 49% reduction in LBR among endometriosis patients with concurrent adenomyosis. These results are consistent with previous meta-analysis and observational studies, suggesting that in cases of coexisting disease, the detrimental impact of adenomyosis on LBR outcomes is more pronounced (30, 58, 59).

**Table 3:** The characteristics of selected studies related to ART outcomes with endometriosis coexisting adenomyosis vice versa.

References	Intervention and/or study type	Comparable and/or positive IVF/ICSI outcomes	Uncertain and/or negative IVF/ICSI outcomes	Conclusion and/or recommendations
Wang et al. (66)	Systematic review and meta-analysis (cohort studies)		Adenomyosis in endometriosis is associated with a significantly LBR (OR = 0.44; 95% CI: 0.26–0.75) than endometriosis alone	In the coexistence of endometriosis and adenomyosis, adenomyosis reduces LBR
Liu et al. (77)	A retrospective analysis	In women with endometriosis and/or adenomyosis, peri-implantation treatment with TNF-α inhibitor increased CPR significantly compared with control group in FET cycles.		More extensive studies are needed.
Rees et al. (79)	A matched retrospective cohort study		the combined adenomyosis and endometriosis group showed a significantly reduced LBR (*p* = 0.001, OR: 0.309 (95% CI: (0.168–0.644))	In the coexistence of endometriosis and adenomyosis, adenomyosis reduces LBR
Sharma et al. (76)	Retrospective cohort study		LBR were 27.47% in controls; 26.48% in women with only endometriosis; 11.36% in women with endometriosis and adenomyosis; and 12.5% in women with only adenomyosis.	In the coexistence of endometriosis and adenomyosis, adenomyosis reduces LBR

CPR, clinical pregnancy rate; LBR, live birt rate; CI, confidence interval.

### Surgery adenomyosis before ART

Adenomyomectomy is the most commonly employed minimally invasive surgical approach for women with adenomyosis. Non-surgical ablative options include high-intensity focused ultrasound (HIFU) and radiofrequency ablation (RFA). In a recent meta-analysis, Liu et al. (81) reported pooled pregnancy rates of 50.1% (95% CI: 40.0–60.2%) following adenomyomectomy and 52.0% (95% CI: 32.4–71.6%) after thermal ablation. Corresponding delivery rates were 39.5% (95% CI: 29.9–49.2%) and 32.5% (95% CI: 26.0–38.9%), respectively. Spontaneous miscarriage rates were 16.3% (95% CI: 9.7–22.9%) after adenomyomectomy and 27.1% (95% CI: 8.1–46.1%) following thermal ablation. Notably, pregnancy loss and miscarriage rates after thermal ablation were relatively higher compared with adenomyomectomy, which was attributed to potential thermal injury to the endometrium. Importantly, none of the included studies were randomized controlled trials.

Uterine artery embolization (UAE) remains controversial in women desiring fertility due to its association with low pregnancy rates, high obstetric complication rates, and impairedendometrial receptivity. A systematic review and meta-analysis by Tan et al. (82) reported uterine rupture and preterm birth rates of 6.8% (3/44) and 4.5% (2/44), respectively, in pregnant women with diffuse adenomyosis following adenomyomectomy, compared with 0% (0/35) and 10.9% (12/110) in those with focal adenomyosis. Only one comparative study demonstrated superior postoperative reproductive outcomes with HIFU (pregnancy rate 52.0%, delivery rate 36.0%) compared with adenomyomectomy (pregnancy rate 30.2%, delivery rate 27.9%) (83). In carefully selected patients, robotic-assisted surgical management may represent a feasible and potentially advantageous option for the treatment of diffuse adenomyosis (84).

### In endometriosis and perinatal outcomes

A meta-analysis by Matsuzaki et al. (85) demonstrated a significantly increased risk of placenta revia in women with severe endometriosis compared with those without endometriosis (OR: 5.22; 95% CI: 2.51–10.85). Among women undergoing IVF/ICSI, the risk of placenta previa was nearly threefold higher when infertility was attributed to endometriosis rather than to other causes (OR: 2.96; 95% CI: 1.25–7.03). Furthermore, endometriosis was also associated with an elevated risk of placenta accreta (adjusted OR: 3.39; 95% CI: 1.96–5.87).

Adenomyosis likewise exerts a negative impact on pregnancy outcomes, with multiple studies reporting an increased risk of preeclampsia (OR range 4.35–7.87), preterm birth (OR: 2.65–3.09), delivery of small-for-gestational-age (SGA) infants (OR: 2.86–3.90), and postpartum haemorrhage (OR: 2.90) (30, 86). Overexpression of oxytocin receptors (OTR) in adenomyosis-affected uteri may lead to uterine hyperperistalsis and microtrauma within the JZ. In this context, administration of an oxytocin receptor antagonist during FET cycles has been suggested to reduce early miscarriage rates in women with adenomyosis (88). Proposed mechanisms underlying adverse pregnancy outcomes in both endometriosis and adenomyosis include increased myometrial prostaglandin production, chronic inflammation, altered uterine contractility, and impaired spiral artery remodelling, ultimately resulting in abnormal placentation. These mechanisms are highly complex and remain a subject of ongoing debate, and potential interactions between the two conditions cannot be excluded. From a phenotypic perspective, a meta-analysis by Xia et al. (89) reported that women with diffuse adenomyosis had significantly higher odds of preterm birth (OR: 1.66; 95% CI: 1.03–2.67; *p* = 0.038) and hypertensive disorders of pregnancy (OR: 2.23; 95% CI: 1.32–3.77; *p* = 0.002).

## Discussion

Endometriosis is associated with a reduced number of retrieved oocytes and high-quality embryos; however, LBR appear to be comparable to those observed in infertility due to other causes (13, 19, 21). Even in women with advanced-stage endometriosis or those with a history of surgical treatment for endometriosis, implantation rates and LBR remain similar to those reported in women with tubal factor infertility. Endometriosis phenotype itself does not significantly influence ART outcomes (13, 14, 19, 22, 24, 28, 29). Importantly, earlier studies may have reported reduced LBR partly due to the inclusion of patients with coexisting adenomyosis, which was not consistently excluded or adequately accounted for in outcome analyses. However, women with endometriosis may still face challenges during IVF/ICSI cycles. Patient age and ovarian reserve, rather than endometriosis *per se*, appear to be the most critical determinants of IVF outcomes in this population. Current evidence indicates that when high-quality embryos are transferred in HRT-FET cycles, no major defects in endometrial receptivity are observed. These findings suggest that endometrial receptivity is comparable regardless of the presence or severity of endometriosis (19). Transcriptomic analyses and data from oocyte donation cycles further support that endometrial receptivity remains normal and is independent of disease stage (19, 27). Accordingly, impaired implantation rates in women with endometriosis may be more closely related to oocyte and embryo quality rather than to endometrial factors. Reduced oocyte yield and compromised embryo quality appear to play an important contributory role in IVF/ICSI success in this population (33). Given that cumulative pregnancy and LBR represent the most relevant outcome measures in IVF/ICSI, the impact of a reduced number of retrieved oocytes has become increasingly important to consider (20, 22, 30, 33). Nevertheless, more comprehensive and well-designed studies are still needed to clarify these associations. In endometriosis, disruption of the ovarian microenvironment through multiple pathophysiological mechanisms, including adverse effects on granulosa cell function, may impair oocyte quality. In addition, diminished ovarian reserve, advanced maternal age, the presence of DIE, and coexisting adenomyosis are all thought to contribute to suboptimal IVF/ICSI outcomes. High body mass index (BMI) has also been shown to exert a negative influence, with obesity adversely affecting LBR (31). Notably, DIE has been identified as a stronger predictor of poor IVF outcomes, with significantly lower pregnancy rates compared with superficial disease (58% vs. 83%) (50).

Adenomyosis and chronic endometritis may potentially impair endometrial receptivity in women with endometriosis (77). Dysregulation of the PI3K/AKT and NOTCH signaling pathways may further compromise implantation by decreasing the expression of key transcription factors associated with endometrial receptivity, including FOXO1 and IGFBP1. Moreover, an increased body mass index (BMI) has been demonstrated to negatively influence live birth rates (LBR) (31). Overall, although some studies report conflicting results, current meta-analyses suggest that IVF outcomes in women with endometriosis are comparable to those of control populations with respect to LBR. The improved exclusion of coexisting adenomyosis through advanced transvaginal ultrasonography (TVUS) and magnetic resonance imaging (MRI) has allowed a more precise evaluation of “pure” endometriosis and facilitated a more accurate interpretation of IVF/ICSI outcomes. However, despite the availability of various therapeutic strategies for endometriosis-associated infertility, standardized treatment protocols prior to IVF/ICSI have not yet been established (21, 30, 32, 33, 35). These findings underscore the importance of individualized patient management in IVF/ICSI, considering disease phenotype, ovarian reserve, age, BMI, and coexisting conditions such as adenomyosis or chronic endometritis.

CPR and LBR following FET of euploid embryos do not differ between women with endometriosis and control populations (27). In addition, compared with fresh ET, FET has been associated with CPR (OR: 1.2), LBR (OR: 1.3), and implantation rates (OR: 1.2) (9). These findings support the concept that endometrial receptivity is preserved regardless of the presence or severity of endometriosis. Conversely, in women with adenomyosis, meta-analyses have demonstrated similar reproductive outcomes between FET and fresh ET strategies (9). Nevertheless, consecutive FET cycles may be considered in women with endometriosis and/or adenomyosis, as cumulative LBR does not appear to be reduced in these patient populations (79). Furthermore, LBRs are not adversely affected by either endometriosis or adenomyosis following the transfer of euploid blastocysts in HRT cycles (19, 60). Collectively, these findings suggest that the suppressive effect of HRT on ovarian function and endogenous hormonal fluctuations may exert a beneficial impact on endometrial receptivity, thereby optimizing implantation and improving LBR in this population.

According to the ESHRE 2022 guidelines, routine surgery prior to ART is not recommended for OMA or SUP (6). Meta-analyses indicate that surgical treatment before IVF/ICSI is associated with reduced LBR per cycle (OR ∼0.53), suggesting potential negative effects on fertility outcomes (31, 46, 50). Although some studies report comparable outcomes between surgery and IVF/ICSI in DIE (47, 48), there is no high-quality RCT evidence demonstrating that operative laparoscopy improves reproductive results (50). Recent meta-analyses show that surgery followed by IVF/ICSI does not significantly improve LBR in OMA (OR: 0.89; 95% CI: 0.68–1.16) or DIE (OR: 1.82; 95% CI: 0.70–4.77), and DIE surgery carries considerable long-term morbidity (complication rates 9%–23%) (35, 51). Therefore, surgery should be reserved for clear clinical indications, and treatment decisions should be individualized. In asymptomatic patients, first-line IVF/ICSI represents an evidence-based approach.

Meta-analyses have consistently shown that adenomyosis is associated with a significant impairment in IVF/ICSI and pregnancy outcomes (30, 57, 58, 62). The meta-analysis by Wang et al. (66) demonstrated that IVF/ICSI outcomes are particularly compromised in women with symptomatic and diffuse adenomyosis. Regardless of phenotypic classification, fertility appears to be adversely affected, especially when adenomyosis involves the JZ (86). In contrast, a study evaluating outcomes after euploid embryo transfer in women with adenomyosis reported comparable CPR, miscarriage rates, and LBR relative to controls (60). However, this finding warrants cautious interpretation, as most patients in that cohort met only a single MUSA criterion, while only 17 patients exhibited two diagnostic features. Notably, the age-adjusted relative risk (aRR) for LBR in women meeting at least one MUSA criterion was 0.58 (95% CI, 0.45–0.75), indicating a substantially reduced likelihood of LBR in the presence of adenomyosis. Moreover, CPR declined markedly with increasing ultrasonographic severity, from 42.7% in women without adenomyosis to 13.0% in those presenting with four to seven ultrasound diagnostic features (61). These severity-dependent effects should be carefully considered when interpreting reproductive outcomes in adenomyosis.

The phenotypic impact of adenomyosis on IVF/ICSI outcomes remains inconsistent. While some studies report an association between specific phenotypes and reduced LBR (53, 66), others show no significant correlation (79), leaving uncertainty regarding the phenotype associated with the poorest reproductive outcomes. Current evidence suggests that JZ involvement may be more strongly associated with impaired fertility outcomes (66, 92, 93). However, studies are highly heterogeneous with respect to patient characteristics, pretreatment strategies, stimulation protocols, and diagnostic criteria. Additional variability arises from phenotypic diversity, frequent coexistence with endometriosis or leiomyomas, and differences in symptom status and imaging modalities (TVUS and/or MRI). Most studies fail to demonstrate a clear association between individual diagnostic features and IVF/ICSI outcomes, limiting comparability and interpretation.

Alson et al. (79) reported that women with coexisting endometriosis and adenomyosis had a 15% lower cumulative probability of achieving a LBR after three consecutive IVF/ICSI cycles compared with women without these conditions. The reduced LBR observed in this subgroup may be attributable to greater adenomyosis severity. Nevertheless, despite the lower cumulative LBR across three IVF/ICSI cycles, women with endometriosis and/or adenomyosis still demonstrate a reasonable probability of achieving a LBR through consecutive treatment cycles (79). In this study, no significant differences in adenomyosis phenotypes were identified between women who achieved pregnancy and those who did not. However, most existing studies have not systematically distinguished between subtypes of endometriosis and adenomyosis. Importantly, the exclusion of coexisting adenomyosis using strict contemporary TVUS and MRI criteria has enhanced the evaluation of “pure” endometriosis in IVF/ICSI studies and enabled a more accurate interpretation of reproductive outcomes.

In adenomyosis, miscarriage appears to occur independently of embryo genetic status. In oocyte donation cycles, implantation rates have been reported to be comparable to those of controls, whereas miscarriage rates remain significantly elevated (64). Similarly, even after the transfer of euploid embryos, miscarriage rates are still increased in women with adenomyosis (65). Furthermore, the endometrial gene expression profile in women with adenomyosis does not significantly differ from that of controls (21). These findings suggest that pregnancy loss associated with adenomyosis may be mediated by molecular mechanisms beyond alterations in genes related to the implantation window. Increased JZ thickness (>10 mm) has been identified as an independent risk factor for implantation failure (63, 64). Cozzolino et al. (86) reported a threefold increase in the relative risk of miscarriage, particularly in cases of diffuse adenomyosis with JZ involvement. Both reduced implantation rates and elevated miscarriage risk appear to be influenced by disease type and extent (71). The age-adjusted relative risk for LBR was most significantly reduced in women with JZ involvement (OR: 0.29, 95% CI: 0.11–0.74) (61). Recent studies further indicate that the predominantly diffuse adenomyosis phenotype is associated with decreased LBR and an increased risk of preterm birth, while JZ involvement negatively impacts fertility outcomes in both focal and diffuse adenomyosis phenotypes (93, 94) (Table 4).

**Table 4:** IVF/ICSI outcomes related to phenotype and junctional zone involvement in adenomyosis.

References	Intervention and/or study type	Comparable and/or positive IVF/ICSI outcomes	Uncertain and/or negative IVF/ICSI outcomes	Junctional zon involvoment	Conclusion and/or recommendations
Trinchant et al. (53)	Multicenter and retrospective cohort study	A higher LBR per transfer was described in *diffuse adenomyosis* compared to focal adenomyosis: 47/166 (28.3%) vs. 9/62 (15%), respectively (OR = 2.32, 95% CI: 1.03–5.78, *p* = 0.034).		NA	*Diffuse adenomyosis* provides a higher LBR
Wang et al. (66)	A systematic review and meta-analysis		Diffuse and symptomatic adenomyosis, lower LBR (OR = 0.57; 95% CI: 0.34–0.96) and miscarriage (OR = 2.48, 95% CI: 1.28–4.82)	NA	*On USG Diffuse and symptomatic* adenomyosis reduces LBR
Bourdon et al. (67)	A single-center observational study (FET vs. Fresh cycle)	LBR (86 (44.1%) vs. 34 (30.6%), respectively), were significantly higher in the freeze-all group compared with the fresh ET group.		NA	The adenomyosis phenotype (*internal diffuse adenomyosis, external focal adenomyosis, and adenomyoma*) was not significantly different between the two groups.
Sharma et al. (92)	A prospective cohort study		Diffuse adenomyosis have a lower LBR (20.65% vs. 29.95%; OR: 0.61, 95% CI: 0.41–0.89, *p* = 0.011) than women without adenomyosis.	Diffuse adenomyotic lesions affecting the JZ exhibited significantly lower LBR (16.42% vs. 25.75%; OR: 0.57, 95% CI: 0.34–0.94, *p* = 0.029)	In diffuse adenomyosis showing JZ involvement, live birth rates are much lower.
Exacoustos et al. (93)	A multicenter, observational, prospective study.			Higher percentage of infertility and miscarriage in focal adenomyosis of both the outer myometrium and the JZ	In focal adenomyosis showing JZ involvement, miscarriae are increased
Cozzolino et al. (86)	A prospective observational cohort study.			Diffuse adenomyosis in the JZ increased the relative risk of miscarriage two-fold (RR, 2.29; 95% CI, 1.22–4.30).	In diffuse adenomyosis showing JZ involvement, miscarriae are increased
Xia et al. (88)	A systematic review and meta-analysis (involving 390 women with diffuse adenomyosis and 233 women with focal adenomyosis)		Diffuse adenomyosis had higher odds of experiencing preterm birth (OR: 1.66, 95% CI: 1.03–2.67, *p* = 0.038) and hypertensive disorders of pregnancy (OR: 2.23, 95% CI: 1.32–3.77; *p* = 0.002).	NA	Diffuse adenomyosis was significantly associated with an increased risk of adverse pregnancy outcomes
Zhu et al. (80)	A retrospective cohort study		LBR decreased by 49 percent.		Adenomyosis was significantly associated with an decreased LBR

NA, not assessed; CBR, clinical pregnancy rate; LBR, live birth rate; JZ, junctional zone; CI, Confidince inreval; FET, frozen embryo cycle.

In adenomyosis, neither adjustment of the ET day based on transcriptomic profiling nor increasing luteal-phase progesterone supplementation has been shown to improve pregnancy outcomes in FET cycles, supporting the concept of progesterone resistance (90). Although some studies suggest that higher luteal progesterone levels may optimize outcomes in endometriosis patients undergoing HRT–FET cycles (91), others report no significant difference in serum progesterone levels between affected and unaffected women (92). Overall, the evidence is conflicting, and the definition of optimal luteal progesterone levels in patients with endometriosis and/or adenomyosis undergoing HRT-FET cycles remains controversial.

Recent meta-analyses indicate promising pregnancy outcomes after surgery for adenomyosis, particularly with uterus-sparing approaches, with rates varying by phenotype and disease extent (80). Uterus-sparing surgical approaches have demonstrated favourable reproductive outcomes, with reported pregnancy rates ranging from 38.5% to 49.1%, depending on the extent and phenotype of adenomyosis (81, 82). Although postoperative GnRH agonist therapy and the combination of surgery with IVF/ICSI may improve outcomes, evidence remains inconsistent. Surgical benefit appears greater in younger patients and those with focal disease, whereas effectiveness is limited in women over 40 years. Given the risk of uterine rupture and uncertain fertility benefit, surgical intervention should be reserved for symptomatic patients with repeated IVF/ICSI failure despite transfer of high-quality embryos (51, 52, 56).

A meta-analysis by Ge Li et al. (68) suggested that the ultra-long GnRH-a protocol may improve fresh ET outcomes in women with adenomyosis, potentially by enhancing implantation efficiency. These findings were supported by an earlier study reporting similar results (71). However, the meta-analysis conducted by Ge et al. (68) had several important limitations, including the absence of RCTs, lack of phenotypic classification of adenomyosis prior to IVF/ICSI, and substantial heterogeneity among the included studies. In this meta-analysis, although CPR were higher and miscarriage rates were comparable in fresh cycles, these improvements did not translate into increased LBR. Consistent with these limitations, the most recent ESHRE guidelines do not recommend the routine use of ultra-long GnRH agonist protocols prior to IVF/ICSI (1, 6). Both endometriosis and adenomyosis are associated with increased obstetric complications. DIE is a recognized risk factor for placenta previa and spontaneous hemoperitoneum during pregnancy. Therefore, disease-specific risks should be considered during antenatal care, and early diagnosis with close monitoring may help reduce adverse maternal and fetal outcomes (83–86, 93).

Endometriosis researchers continue to express concern that advancements in patient care remain limited, largely owing to the complexity of the clinical manifestations and an incomplete understanding of the underlying pathophysiological mechanisms driving these symptoms. The prediction of natural fertility outcomes in women with endometriosis remains problematic. Moreover, concomitant adenomyosis may contribute to reduced IVF success in women with endometriosis. Endometriosis and adenomyosis, either independently or in combination, negatively influence pregnancy and delivery outcomes. The coexistence of both conditions is associated with a more pronounced reduction in fertility potential, and the probability of achieving spontaneous conception decreases further when they occur together. Whereas endometriosis is linked to a prolonged time to spontaneous conception, adenomyosis is more frequently associated with an increased risk of early pregnancy loss. Both disorders have also been correlated with adverse obstetric outcomes, including placenta previa, preterm birth, and higher rates of cesarean delivery. With an average diagnostic delay of seven years, endometriosis is usually diagnosed during reproductive age, often coinciding with the early phase of a woman's professional life (4, 5). Age is the most important prognostic factor in IVF outcomes. Early and accurate diagnosis is critical for guiding appropriate therapeutic management, with medical hormone therapy representing the first-line treatment strategy. Treatment adherence may be enhanced through regular longitudinal follow-up and individualized adjustment of hormonal regimens according to patient-specific clinical characteristics. In addition to improving quality of life, medical therapy may contribute to limiting disease progression in terms of extent and severity. Long-term surveillance facilitates monitoring of treatment compliance and is associated with improved clinical outcomes (33, 37, 87, 94).

## Summary

LBR after IVF/ICSI are comparable between women with isolated endometriosis and those without disease; therefore, routine laparoscopic surgery prior to treatment is not recommended except in symptomatic patients. However, DIE and concomitant adenomyosis may negatively affect IVF/ICSI outcomes, and the surgical management of DIE before ART remains controversial. In asymptomatic patients, IVF/ICSI should be considered the first-line approach.

The impact of adenomyosis on reproductive outcomes is heterogeneous. JZ disruption, symptomatic and diffuse phenotypes, increased uterine volume, and greater disease severity are associated with impaired implantation and higher miscarriage rates. The efficacy of GnRH analogue pretreatment remains uncertain, with inconsistent results for both fresh and frozen embryo transfer cycles. While FET may improve outcomes in endometriosis, comparable results have been reported for fresh and frozen transfers in adenomyosis. Overall, adenomyosis coexisting with endometriosis significantly worsens IVF/ICSI outcomes, particularly by reducing LBR and increasing miscarriage risk. Individualized management based on disease phenotype, symptom severity, age, and prior ART response is essential, and both conditions are associated with increased obstetric complications requiring close monitoring. Data regarding any of the subjects in the study has not been previously published unless specified. Data will be made available to the editors of the journal pre and/or post publication for review or query upon request.
